# m^1^A‐Dependent TRMT6/61A‐ARG2 Axis Drives Protumorigenic Senescence by Remodeling the Tumor Microenvironment

**DOI:** 10.1002/advs.202518536

**Published:** 2026-01-08

**Authors:** Tuoyang Li, Mingzhe Huang, Jinlin Cai, Xuan Li, Yaokang Xing, Rongzhao He, Zixiao Wan, Yingguo Gan, Ziming Li, Jingrong Weng, Yumo Xie, Yuanhui Wu, Xiaoxia Liu, Xiaolin Wang, Yanxin Luo, Meijin Huang, Jinxin Lin, Huichuan Yu

**Affiliations:** ^1^ Department of General Surgery (Colorectal Surgery) The Sixth Affiliated Hospital Sun Yat‐sen University Guangzhou China; ^2^ Guangdong Institute of Gastroenterology Guangzhou China; ^3^ Guangdong Provincial Key Laboratory of Colorectal and Pelvic Floor Diseases The Sixth Affiliated Hospital Sun Yat‐sen University Guangzhou China; ^4^ Biomedical Innovation Center The Sixth Affiliated Hospital Sun Yat‐sen University Guangzhou China; ^5^ Key Laboratory of Human Microbiome and Chronic Diseases (Sun Yat‐sen University) Ministry of Education Guangzhou China; ^6^ Sun Yat‐sen University Institute of Advanced Studies Hong Kong Hong Kong China

**Keywords:** colorectal cancer, m^1^A, senescence‐associated secretory phenotype, senescent tumor cells, tumor microenvironment

## Abstract

Cellular senescence, a fundamental hallmark of aging, plays a paradoxical, often pro‐tumorigenic role in cancer. This malignancy is largely driven by the senescence‐associated secretory phenotype (SASP), yet the mechanisms that govern the production of a pro‐tumorigenic SASP remain poorly understood. This study uncovers an epitranscriptomic axis in colorectal cancer (CRC) where the TRMT6/TRMT61A tRNA *N* ^1^‐methyladenosine (m^1^A) methyltransferase complex is aberrantly elevated, driving a senescent state in malignant cells. Mechanistically, TRMT6/61A‐dependent m^1^A deposition on specific tRNAs enhances the translational efficiency of their cognate codons. This codon‐biased translational control selectively boosts the synthesis of ARG2. Accumulation of ARG2 subsequently activates mTOR and NF‐κB signaling and thereby establishes a robust SASP, which actively reprograms the tumor microenvironment by promoting the growth and invasiveness of neighboring cancer cells, activating cancer‐associated fibroblasts, and polarizing immunosuppressive M2 macrophages. Collectively, these findings define the TRMT6/61A‐ARG2 pathway as a driver for pro‐tumorigenic senescence in an m^1^A‐dependent manner, revealing a new layer of translational control in aging‐associated pathology and offering a compelling rationale for developing senomorphic therapies.

## Graphical abstract

Uncovering a new layer of translational control, this study reveals how TRMT6/TRMT61A‐mediated tRNA‐m^1^A methylation drives pro‐tumorigenic senescence in colorectal cancer. By selectively enhancing ARG2 translation, this epitranscriptomic axis triggers an NF‐κB‐dependent SASP. This program actively remodels the tumor microenvironment to promote malignancy, identifying the TRMT6‐ARG2 axis as a potent target for senomorphic therapy.

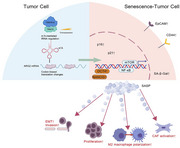

## 1 Introduction

Cellular senescence has been observed in tumor, epithelial, endothelial, stromal, and immune cells in cancer tissues [1]. Among the senescent populations, the role of senescent non‐tumor cells in cancer progression has been well understood, and they could promote cancer development by secreting a cocktail of inflammatory factors known as senescence‐associated secretory phenotype (SASP), which fundamentally remodels the tumor microenvironment (TME) to promote immune evasion, angiogenesis, and invasion [2, 3, 4]. Recently, the cancer‐promoting feature of senescent tumor cells (STCs) is continuously being discovered, while the role and mechanism of STCs production and its crosstalk with other cells in TME remain unknown [1].

While senescence in tumor cells initially acts as a tumor‐suppressive barrier by arresting cell proliferation, our understanding has evolved to recognize its pro‐tumorigenic role within TME [5, 6, 7]. The spontaneous emergence of these STCs is a documented feature of CRC and other malignancies, suggesting that they represent a critical, yet poorly understood, reservoir for disease progression and therapeutic resistance. Therefore, deciphering the molecular switches that not only induce this senescent state but also dictate its pro‐malignant SASP profile is urgently needed for the development of novel therapies.

The transition to a pro‐tumorigenic senescent state is driven by extensive senescence‐associated reprogramming. This involves the modulation of complex signaling cascades and profound epigenetic shifts. While the roles of DNA methylation and histone modifications in silencing pro‐proliferative genes are well‐established [8, 9], recent studies have begun to uncover the critical involvement of post‐transcriptional RNA modifications. For instance, *N* ^6^‐methyladenosine (m^6^A) has been shown to regulate the stability and translation of key mRNAs involved in the senescence program [10]. However, the full landscape of RNA modifications that dictate the functional plasticity of STCs, particularly those controlling the selective translation of proteins that shape the TME, remains largely unexplored.

*N* ^1^‐methyladenosine (m^1^A) is a dynamic post‐transcriptional RNA modification that regulates translation efficiency and fidelity by influencing tRNA stability and ribosome interactions [11, 12]. The primary enzymatic “writer” of m^1^A on tRNA, the TRMT6/TRMT61A complex, has been implicated in diverse biological processes [13, 14, 15], and its dysregulation is observed in several cancers [16, 17]. Yet, its contribution to STCs' persistence and the specific regulation of the SASP remains elusive. We hypothesized that TRMT6/61A‐mediated tRNA‐m^1^A modification could act as a translational rheostat, selectively enhancing the synthesis of key effector proteins, such as arginase 2 (ARG2), thereby orchestrating a pro‐tumorigenic SASP and shaping a cancer‐promoting TME.

In this study, we elucidate the TRMT6/61A‐ARG2 axis as a driver of pro‐malignant senescence in CRC. Our findings reveal how a specific epitranscriptomic event reshapes the tumor microenvironment, providing a novel insight into RNA‐modification‐based cancer therapeutics.

## 2 Results

### 2.1 Aberrant Elevation of RNA m^1^A Methylation in CRC Tissues

**FIGURE 1:**
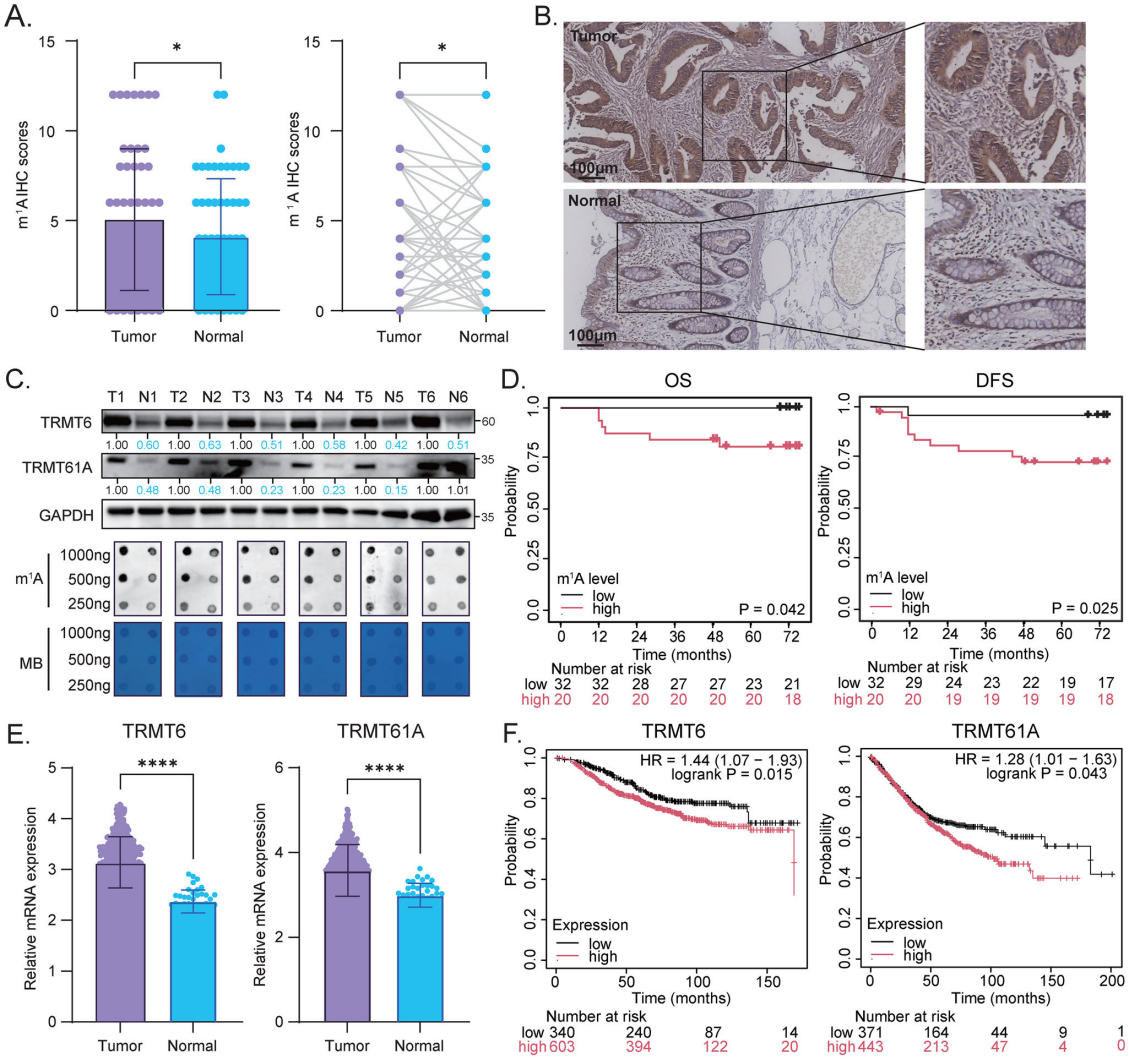
RNA m^1^A methylation is aberrantly elevated in colorectal cancer tissues and correlates with poor prognosis. (A) Overall (left) and paired (right) comparison of immunohistochemistry (IHC) scores for RNA m^1^A abundance in paired colorectal cancer (CRC) tumor (T) and adjacent normal (N) tissues from the NEPDC cohort (*n* = 58). The IHC score (Immunoreactive Score, IRS) was calculated by multiplying the staining intensity score (0–3) by the percentage of positive cells score (0–4). (B) Representative IHC images showing more abundant m^1^A in CRC tumor tissue compared to its adjacent normal tissue. Scale bar, 100 µm. (C) Blot assays for TRMT6/TRMT61A protein expression (top) and global m^1^A levels (bottom) in six pairs of CRC tumor (T) and adjacent normal (N) tissues. The dot blot assay (bottom) loaded decreasing amounts of total RNA (1000 ng, 500 ng, and 250 ng) probed with an anti‐m^1^A antibody, and the methylene blue (MB) staining served as a loading control for RNA. Relative densitometry quantification (normalized to GAPDH) is shown below the bands. Blots are representative of three independent biological replicates (*n* = 3). (D) Kaplan‐Meier analysis of overall survival (OS) and disease‐free survival (DFS) outcomes in the NEPDC cohort based on m^1^A IHC scores. Patients were stratified into high and low m^1^A level groups using the median IRS value as the cutoff, and the *p*‐values were calculated using the log‐rank test. (E) External validation of significantly higher expression of both *TRMT6* and *TRMT61A* in tumor tissues compared to normal tissues using public CRC datasets. (F) Kaplan‐Meier analysis showed that the higher mRNA expression of *TRMT6* and *TRMT61A* was associated with worse OS in the external validation cohort. All the data were presented as mean ± SD. ^*^ *p* < 0.05, ^**^ *p* < 0.01, ^***^ *p* < 0.001, ^****^ *p* < 0.0001.

In the National Basic Research Program of Evolution from Precancerous Disease to Cancer in China (NEPDC) Quantitative real‐time PCR cohort, the immunohistochemistry (IHC) and dot blot assays revealed a significant increase in m^1^A levels within the tumor tissues compared to the adjacent normal tissues (Figure 1A–C). In addition, patients with higher m^1^A methylation levels exhibited worse survival outcomes (Figure 1D). Expectedly, the Western blot assay showed an elevated expression of the m^1^A writer, the TRMT6/61A complex, in tumor tissues (Figure 1C). This observation was further corroborated by analyzing public cancer genomics datasets. We observed an upregulation of m^1^A writers, including *TRMT6*, *TRMT61A*, *TRMT61B*, and *TRMT10C*, in tumor tissues compared to normal tissues (Figure 1E; Figure S1A). Among them, elevated expression of *TRMT6* and *TRMT61A* was significantly associated with worse survival outcomes (Figure 1F; Figure S1B). Our findings indicated an aberrant elevation of the TRMT6/61A complex and m^1^A methylation in CRC tissues, which was associated with poor survival outcomes.

### 2.2 TRMT6/61A‐Mediated m^1^A Modification Drives Senescence in Tumor Cells

**FIGURE 2:**
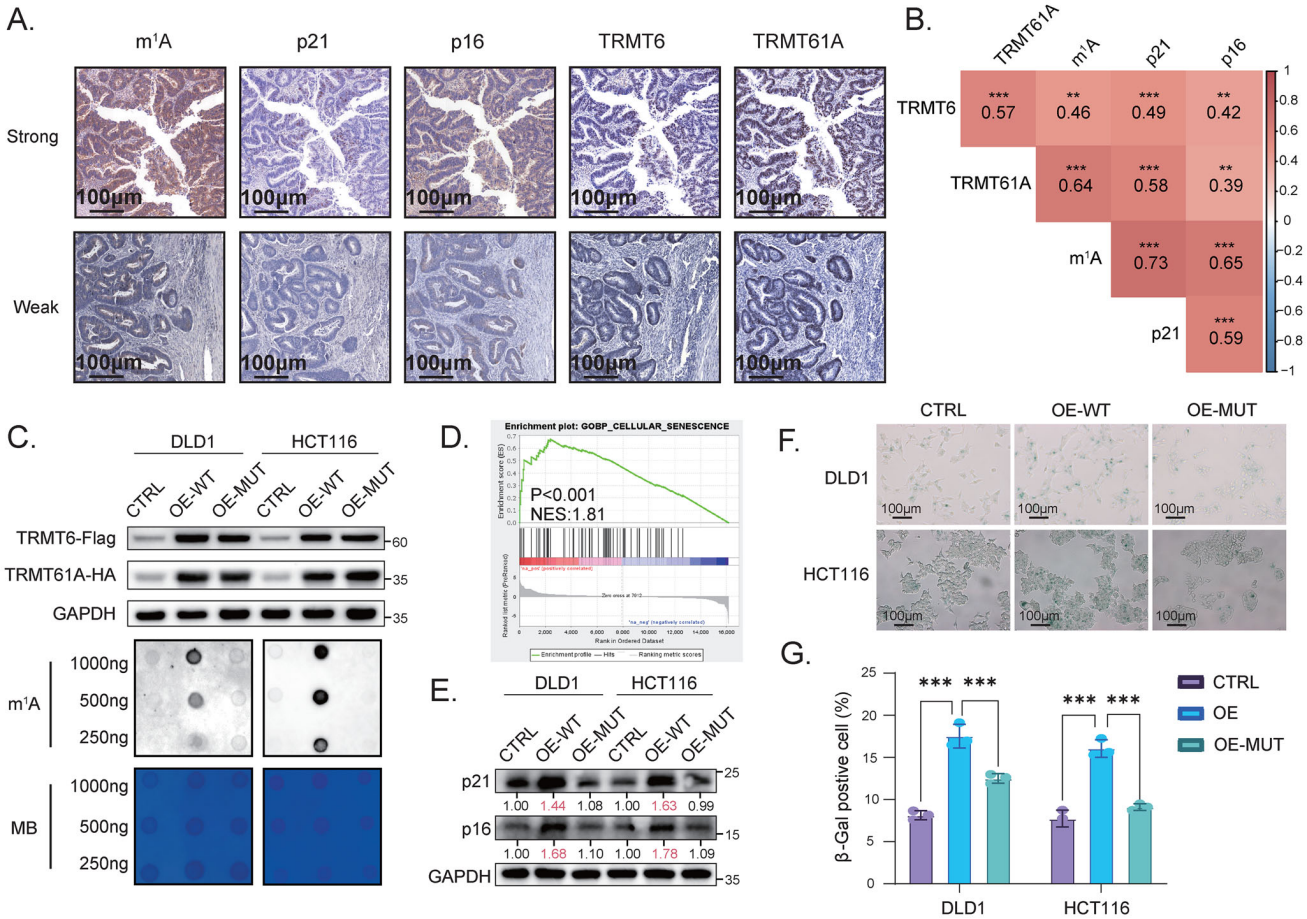
TRMT6/TRMT61A‐mediated m^1^A modification drives cellular senescence in CRC. (A) Representative immunohistochemistry (IHC) images showing co‐expression patterns of m^1^A, p21, p16, TRMT6, and TRMT61A in CRC tumor tissues. The upper and lower rows displayed the two regions with highly and lowly concurrent staining of three markers, respectively. Scale bars, 100 µm. (B) Pearson's correlation heatmap that visualized the relationships between the IHC scores of intracellular markers, including TRMT6, TRMT61A, m^1^A, p21, and p16 in CRC tumor tissues (NEPDC cohort, *n* = 58) was shown. The numbers in the boxes represented Pearson correlation coefficients (r), and the color scale indicated the strength of the correlation. (C) The TRMT6/61A‐mediated RNA m^1^A modification depended on the catalytic domains. We constructed the DLD1 and HCT116 cells with stable overexpression of vector (CTRL), wild‐type TRMT6/61A (OE‐WT), and catalytically inactive mutant TRMT6‐R377L/TRMT61A‐D181A (OE‐MUT) (top), and the dot blot analysis showed global RNA m^1^A levels were elevated in OE‐WT cells rather than OE‐MUT cells compared with CTRL cells (bottom). The methylene blue (MB) staining served as a loading control for RNA. (D) Gene set enrichment analysis (GSEA) of differentially expressed genes between OE‐WT and CTRL groups of DLD1 cells based on their RNA‐seq profiles. The plot showed that cellular senescence was active in the cells highly expressing wild‐type TRMT6/61A. NES: Normalized enrichment score. (E) Western blot analysis demonstrating increased protein levels of senescence markers p21 and p16 in the OE‐WT rather than the OE‐MUT group of DLD1 and HCT116 cells compared with the CTRL group cells. Relative densitometry quantification (normalized to GAPDH) is shown below the bands. Blots are representative of three independent biological replicates (*n* = 3). (F) Representative images of senescence‐associated β‐galactosidase (SA‐β‐Gal) staining in the three groups of DLD1 and HCT116 cells. The senescent cells were identified by their blue color. Scale bars, 100 µm. (G) Proportions of SA‐β‐Gal‐positive cells in the three groups of DLD1 and HCT116 cells from three independent experiments. The percentage of SA‐β‐Gal‐positive cells was determined by quantifying both positive (blue‐stained) and total cells from three randomly selected high‐power fields (100×) per well using ImageJ software. Data are presented as mean ± SD from three independent experiments. ^*^ *p* < 0.05, ^**^ *p* < 0.01, ^***^ *p* < 0.001, ^****^ *p* < 0.0001.

Consistent with prior studies demonstrating spontaneous senescence in tumor cells [18], we investigated the relationship between m^1^A methylation and STCs. IHC analysis of CRC tissues revealed elevated m^1^A methylation levels in regions co‐expressing the senescence markers p21 and p16 (Figure 2A). Notably, quantitative analysis of serial sections from the NEPDC cohort (*n* = 58) revealed a striking concordance. As visualized in the correlation heatmap (Figure 2B), we observed distinct positive correlations between the levels of methyltransferase complex components (TRMT6 and TRMT61A) and global RNA m^1^A abundance. Furthermore, m^1^A levels exhibited significant positive correlations with the canonical senescence markers p21 and p16, suggesting that the TRMT6/61A‐m^1^A axis is a prevalent and clinically relevant pathway associated with senescence in human CRC.

To determine the role of TRMT6/61A‐mediated m^1^A methylation in regulating STCs' phenotype, we generated DLD1 and HCT116 CRC cell lines stably overexpressing wild‐type TRMT6/61A (T6‐OE‐WT) or catalytically inactive mutants (T6‐OE‐MUT) (Figure 2C). The dot blot assay showed that the m^1^A methylation level was elevated in the T6‐OE‐WT cells compared with the vector group, while it remained unchanged in the T6‐OE‐MUT cells, which suggested a TRMT6/61A‐mediated upregulation of m^1^A methylation in T6‐OE‐WT cells and catalytically inactive methyltransferase in T6‐OE‐MUT cells (Figure 2C). We leveraged these models to investigate if TRMT6/61A regulated senescence in an m^1^A‐dependent manner. The gene set enrichment analysis (GSEA) based on RNA‐seq revealed an active biological process involved in cell senescence in T6‐OE‐WT cells (Figure 2D). In addition, both the expression of p21 and p16 and SA‐β‐Gal activity were significantly increased in the OE‐WT cells, while they were not increased in the T6‐OE‐MUT cells (Figure 2E–G). These results indicated that TRMT6/61A‐driven senescence depends on its enzymatic activity to install m^1^A modifications.

### 2.3 TRMT6/61A‐Mediated tRNA‐m^1^A Methylation Drives Senescence by Enhancing ARG2 Translation

**FIGURE 3:**
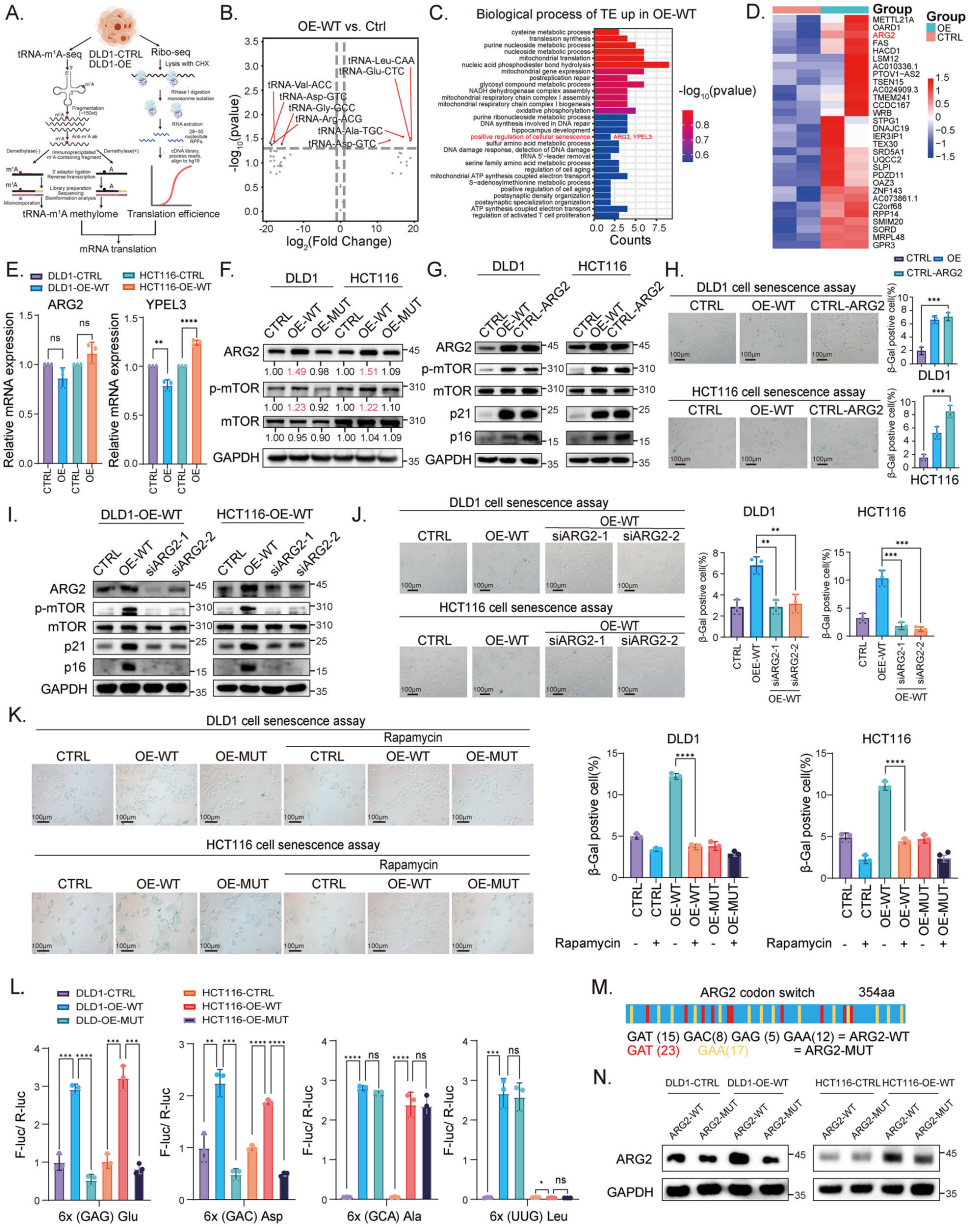
TRMT6/TRMT61A‐mediated m^1^A modification promotes cellular senescence by enhancing the translational efficiency of ARG2 (A) Schematic diagram illustrating the tRNA m^1^A‐seq workflow. (B) Scatter plot of differentially m^1^A‐modified tRNA between DLD1 cells with stable overexpression of vector (CTRL) and wild‐type TRMT6/61A (OE‐WT) based on their tRNA‐m^1^A‐seq profiles. Red dots represented tRNAs with significantly upregulated m^1^A methylation levels (Fold Change > 1 and *p* < 0.05) in the OE‐WT group. (C) Gene ontology (GO) enrichment analysis of transcripts with upregulated translation efficiency in OE‐WT cells compared to CTRL cells, as determined by ribosome profiling (Ribo‐seq). The enriched biological processes were ranked by *p*‐value, and the positive regulation of cellular senescence involving *ARG2* and *YPEL3* was highlighted. (D) A heatmap illustrating the top 30 genes with the highest increase in translation efficiency in OE‐WT cells, and the senescence‐associated gene, *ARG2*, was highlighted. (E) RT‐qPCR analysis of *ARG2* and *YPEL3* mRNA expression in the CTRL and OE‐WT cells. Data were presented as scatter dot plots showing individual data points from three independent experiments (*n* = 3), along with mean ± SD. (F) Western blot analysis showed that the protein levels of ARG2 and phosphorylated mTOR (p‐mTOR) were increased in OE‐WT cells rather than OE‐MUT cells, compared with CTRL cells. Relative densitometry quantification (normalized to GAPDH) is shown below the bands. Blots are representative of three independent biological replicates (*n* = 3). (G, H) Overexpression of ARG2 (OE‐ARG2) induced cellular senescence similar to wild‐type TRMT6/61A. (G) Western blot analysis confirmed ARG2 overexpression and mTOR activation. (H) SA‐β‐Gal staining images and quantification. The percentage of positive cells was quantified from three random fields. (I, J) Knockdown of *ARG2* rescued TRMT6/61A‐induced senescence. The Western blot showed that ARG2 knockdown reversed the increased levels of p‐mTOR, p21, and p16 in OE‐WT HCT116 and DLD1 cells (I), and the decreased SA‐β‐Gal‐positive cells after ARG2 knockdown in OE‐WT cells with representative images were shown (J). (K) The mTOR pathway is required for TRMT6/61A‐induced senescence. Each group of cells was treated with the mTOR inhibitor rapamycin for 48 h. Representative images and quantification of SA‐β‐Gal staining showed that rapamycin treatment reversed the senescent phenotype in OE‐WT cells. Scale bars, 100 µm. (L) The dual‐luciferase reporter system. A schematic diagram of the reporter constructs is provided in Figure S1D. We constructed reporter plasmids by inserting six‐repeated cognate codons for either Asp (GAC) or Glu (GAG)—decoded by the m^1^A‐hypermethylated tRNA‐Asp‐GTC and tRNA‐Glu‐CTC, respectively—immediately upstream of the firefly luciferase gene. The relative luciferase activity was measured. The enhanced ratio in T6‐OE‐WT cells demonstrated that TRMT6/61A‐mediated m^1^A modification of these tRNAs directly enhanced the translational efficiency of their cognate codons. (M) Schematic diagram of the ARG2 codon‐switch assay. To validate specificity, we engineered a synonymous mutant of ARG2 (ARG2‐MUT) by replacing the key cognate codons (GAC and GAG) with different, synonymous codons not recognized by the m^1^A‐modified tRNAs. aa, amino acid. (N) Western blot analysis comparing the protein expression of wild‐type ARG2 (ARG2‐WT) and the codon‐mutated version (ARG2‐MUT) in CTRL and OE‐WT cells, demonstrating that translation of ARG2‐WT is preferentially enhanced in OE‐WT cells. ^*^ *p* < 0.05, ^**^ *p* < 0.01, ^***^ *p* < 0.001, ^****^ *p* < 0.0001.

Having shown that global m^1^A levels were elevated in STCs, we then sought to interrogate differentially methylated tRNAs. To this end, we performed tRNA‐m^1^A‐seq in T6‐OE‐WT and vector group of DLD1 cells to map the TRMT6/61A‐mediated tRNA‐m^1^A atlas at a single‐nucleotide resolution (Figure 3A). The differentially methylated tRNA analysis revealed significant m^1^A hypermethylation in four tRNAs, including tRNA‐Asp‐GTC, tRNA‐Ala‐TGC, tRNA‐Glu‐CTC, and tRNA‐Leu‐CAA (Figure 3B). To further identify the transcripts whose translation was regulated by these m^1^A‐elevated tRNAs, we performed ribosome profiling sequencing (Ribo‐seq) in T6‐OE‐WT and vector group of DLD1 cells. Gene ontology (GO) analysis of transcripts with elevated translation efficiency (TE) in OE‐WT cells highlighted enrichment in cellular senescence (Figure 3C). To determine if this translational enhancement was a global phenomenon driven simply by codon count, we analyzed the correlation between the frequency of cognate codons (GAG and GAC) and TE fold‐change across the transcriptome. As shown in Figure S1C, while TRMT6/61A overexpression induced a significant global remodeling of the translatome, there was no simple linear correlation between codon frequency and TE (*r* = −0.058). This suggests that TRMT6/61A‐mediated tRNA‐m^1^A methylation selectively enhances the translation of target genes such as *ARG2*.

Among the TE‐elevated transcripts, *ARG2* was both a top‐ranked TE‐enhanced transcript (Figure 3D) and a key gene involved in TRMT6/61A‐mediated cell senescence (Figure 3C). Given prior evidence that mTOR signaling drives senescence and is activated by ARG2 [19], we hypothesized that TRMT6/61A may promote senescence via m^1^A‐dependent ARG2 synthesis. As expected, *ARG2* mRNA abundance remained unchanged between the two groups (Figure 3E), while the Western blot analysis confirmed a significant increase in ARG2 protein levels in T6‐OE‐WT cells (Figure 3F). In addition, phospho‐mTOR (Ser2448) levels were increased in T6‐OE‐WT cells, suggesting an activation of the mTOR pathway in the T6‐OE‐WT cells with ARG2 accumulation (Figure 3F).

Next, we sought to investigate whether TRMT6/61A regulated STCs through ARG2. The overexpression of ARG2 in the vector group of DLD1 and HCT116 cells markedly induced cellular senescence, as demonstrated by increased protein levels of p21, p16, and phospho‐mTOR, along with elevated SA‐β‐Gal‐positive cell proportions (Figure 3G,H), which were consistent with the results in the T6‐OE‐WT and vector group of cells (Figure 3I). Mechanistically, the knockdown of *ARG2* in T6‐OE‐WT cells completely blocked the activation of mTOR signaling (Figure 3I), suggesting that ARG2 is required for mTOR activation in this axis. Consequently, the senescent phenotype was rescued after a knockdown of *ARG2* in T6‐OE‐WT DLD1/HCT116 cells (Figure 3I,J). Functional validation using the mTOR inhibitor rapamycin revealed that suppression of the mTOR pathway abolished TRMT6/61A‐induced cell senescence. SA‐β‐Gal‐positive cells decreased in OE‐WT cells treated with rapamycin compared to vehicle controls (Figure 3K). These results confirmed that ARG2 could drive senescence in tumor cells, and TRMT6/61A‐induced senescence depended on ARG2.

Considering that m^1^A in tRNA was reported to promote translation [20], we further tested if the TRMT6/61A‐mediated tRNA‐m^1^A could improve TE. A schematic diagram of the reporter constructs was provided in Figure S1D. We found that TRMT6/61A overexpression enhanced the translation levels of tRNA‐Asp‐GTC and tRNA‐Glu‐CTC (Figure 3L). To validate the role of m^1^A‐modified tRNAs in ARG2 synthesis, we engineered synonymous mutations in *ARG2* codons decoded by tRNA‐Asp‐GTC and tRNA‐Glu‐CTC (Figure 3M). In T6‐OE‐WT cells, wild‐type ARG2 (ARG2‐WT) represented higher protein expression than the codon‐optimized mutant (ARG2‐MUT) (Figure 3N). This demonstrated that m^1^A methylation at specific tRNAs directly enhanced the *ARG2* translation. Together, TRMT6/61A‐induced senescence depended on the m^1^A‐mediated translational enhancement of ARG2.

### 2.4 TRMT6/61A‐Driven Senescence is Predicted to Remodel the TME via a Pro‐Tumorigenic SASP

**FIGURE 4:**
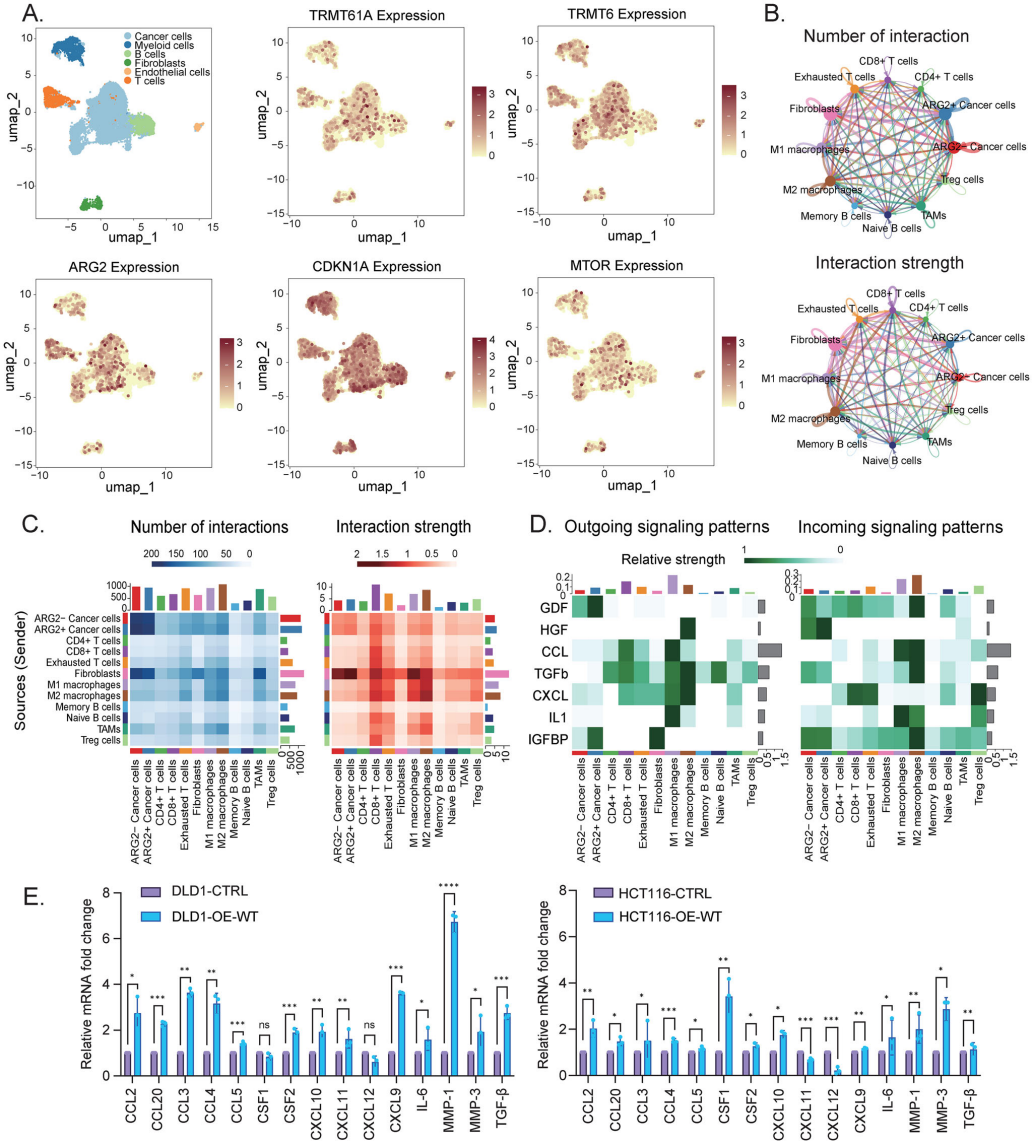
Senescent tumor cells reprogram the tumor microenvironment in CRC (A) Uniform manifold approximation and projection (UMAP) visualization of integrated single‐cell transcriptomes from the CRC patients. The cell clusters with annotation of major cell types were shown (left top), and the feature plots showed the expression of *TRMT6*, *TRMT61A*, *ARG2, CDKN1A*, and *MTOR* among cell types. (B) Chord diagrams illustrating the predicted intercellular communication network based on the number of interactions (top) and the overall interaction strength (bottom). Tumor cells were sub‐classified into *ARG2*+ and *ARG2*‐ subsets to reveal their distinct communication patterns. (C) Heatmaps provided an alternative visualization of the data in (B), quantifying the number of interactions (left) and interaction strength (right) between all identified cell populations. (D) Heatmaps visualizing the outgoing and incoming signaling patterns for key SASP‐related pathways, including GDF, HGF, CCL, TGFb, CXCL, IL1, and IGFBP. (e.g., HGF and IGFBP). The analysis identified *ARG2*+ cancer cells as prominent sources of these signals targeting M2 macrophages and fibroblasts. (E) In vitro validation of SASP gene expression. RT‐qPCR analysis revealed the relative mRNA fold change of a panel of key SASP factors in DLD1 (left) and HCT116 (right) cells. Expression in the TRMT6/61A‐overexpressing group (OE‐WT) is compared to the control group (CTRL). Data were presented as mean ± SD from three independent biological replicates (*n* = 3). ^*^ *p* < 0.05, ^**^ *p* < 0.01, ^***^ *p* < 0.001, ^****^ *p* < 0.0001.

Having established that TRMT6/61A drives cellular senescence in vitro (Figures 2 and 3), we next sought to understand its relevance within the context of TME. We analyzed public single‐cell RNA sequencing data to explore the interplay between senescent‐like tumor cells and other TME components in an unbiased manner. The uniform manifold approximation and projection (UMAP) identified distinct cell populations, including tumor cells that co‐expressed *TRMT6*, *TRMT61A*, *ARG2*, and the senescence effectors *CDKN1A* and *MTOR* (Figure 4A). Notably, feature plots confirmed that the expression of the m^1^A regulators *TRMT6* and *TRMT61A*, along with their key downstream target *ARG2*, was prevalently enriched within the cancer cell cluster. Analysis of individual tumors confirmed this was a common feature across the patient cohort, with a certain proportion of cancer cells expressing these genes in each sample (Figure S1E). Consistent with our proposed mechanism, the expression of the senescence marker *CDKN1A* and *MTOR* was prevalently observed in the cancer cells expressing *TRMT6*, *TRMT61A*, and *ARG2* (Figure 4A). In addition, the differential gene expression analysis highlighted that *TRMT6*, *TRMT61A*, and SASP factors, including *CCL20* and *GDF15*, were upregulated in cancer cells (Figure S1F).

To generate hypotheses about intercellular crosstalk, we performed communication network analysis, which predicted a dense network of interactions between *ARG2*‐positive (*ARG2*+) cancer cells and other key TME players, particularly M2 macrophages and fibroblasts. The analysis of intercellular communication revealed a complex landscape of interactions involving cancer cells, M2 macrophages, and fibroblasts (Figure 4B). Quantification of these interactions confirmed that cancer cells, M2 macrophages, and fibroblasts were among the most highly interacting cell types in terms of both number and strength of connections (Figure 4C). Further dissection of SASP‐related signaling pathways (e.g., GDF and IGFBP) showed that *ARG2*+ cancer cells acted as significant sources of outgoing signals involved in these pathways (Figure 4D, left panel). Critically, M2 macrophages and fibroblasts were identified as key recipients of these SASP signals emanating from *ARG2*+ cancer cells. For example, *ARG2*+ cancer cells exhibited prominent IGFBP signaling toward *ARG2*+ cancer cells, M2 macrophages, and fibroblasts, as well as robust GDF signaling toward *ARG2*+ cancer cells and M2 macrophages (Figure 4D, right panel).

These analyses strongly suggest that TRMT6/61A‐driven senescent tumor cells may actively remodel the TME through secreted factors. To build a model to functionally test this hypothesis, we first needed to characterize the secretome of our T6‐OE‐WT senescent cells. Indeed, Quantitative real‐time PCR (RT‐qPCR) analysis confirmed that T6‐OE‐WT cells exhibited significantly increased expression of a broad panel of SASP factors, including *CCL2*, *CCL20*, *IL‐6*, and *TGF‐β* (Figure 4E). Notably, these factors are known downstream targets of the NF‐κB pathway and have been previously reported to promote tumor cell aggressiveness, induce M2 macrophage polarization, and activate cancer‐associated fibroblasts (CAFs).

### 2.5 TRMT6/61A‐Induced STCs Promote the Malignant Progression of Recipient Cancer Cells through SASP

**FIGURE 5:**
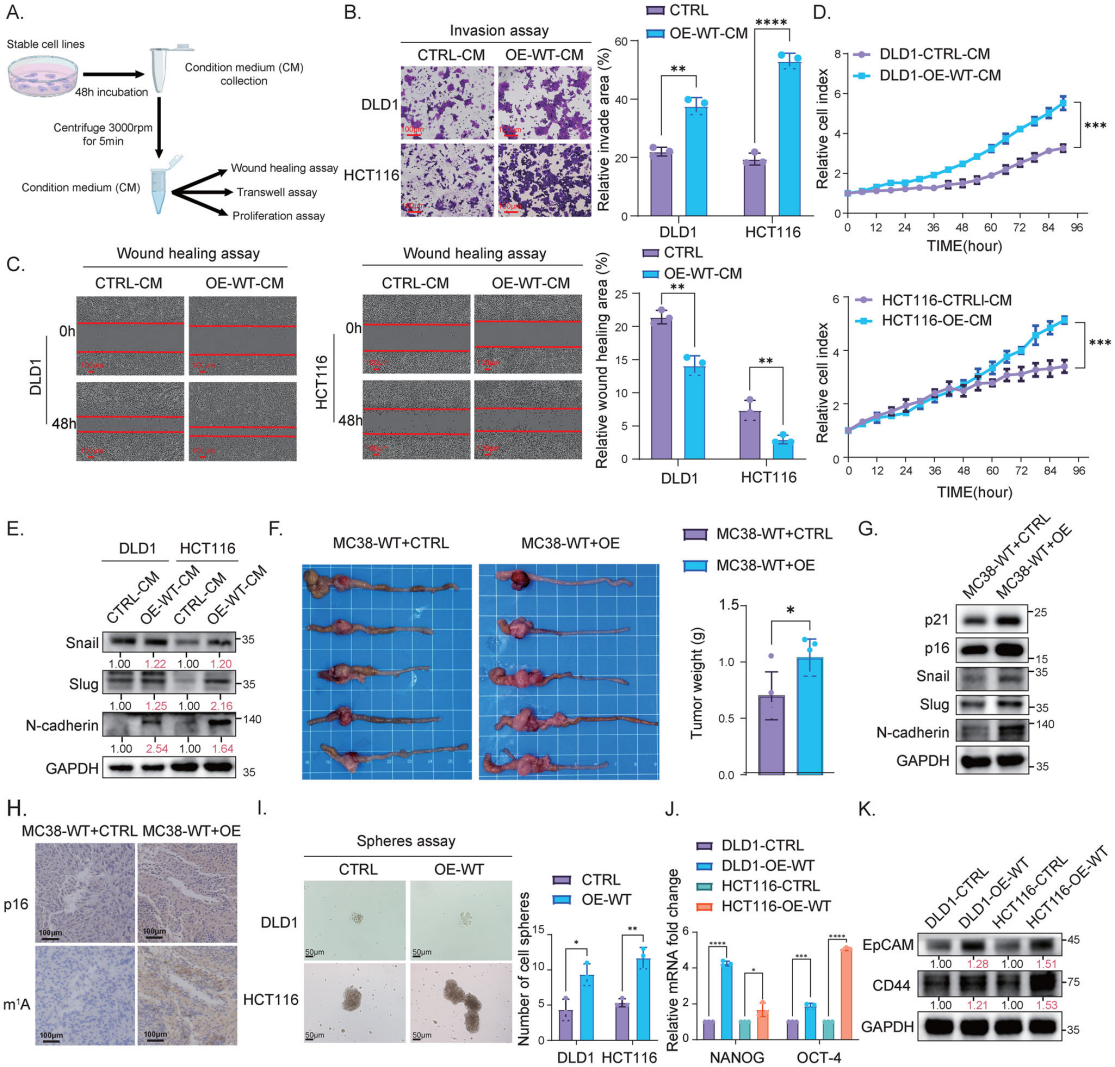
TRMT6/61A‐induced STCs promote the malignant biological behaviors of recipient cancer cells. (A) Schematic diagram illustrating the experimental design. Conditioned medium (CM) was collected from cancer cells with stable overexpression of vector (CTRL) and wild‐type TRMT6/TRMT61A (OE‐WT), respectively. The two groups of CM were subsequently used to culture non‐transfected recipient CRC cells for functional assays. (B, C) The CM from OE‐WT cells enhanced the migration and invasion of recipient CRC cells. Representative images and quantification of transwell invasion (B) and wound‐healing (C) assays for DLD1 and HCT116 cells cultured with the two groups of CM are shown. Scale bars for (B), 100 µm. (D) CM from OE‐WT cells promoted the proliferation of recipient CRC cells. Real‐time cell proliferation curves for DLD1 and HCT116 cells were generated using the IncuCyte system. (E) Western blot analysis showing that CM from OE‐WT cells induced epithelial‐mesenchymal transition (EMT) in recipient DLD1 and HCT116 cells, as indicated by the upregulation of Snail, Slug, and N‐cadherin. Relative densitometry quantification (normalized to GAPDH) is shown below the bands. Blots are representative of three independent biological replicates (*n* = 3). (F) TRMT6/61A‐overexpressing cancer cells promoted tumor growth in an in vivo model. The MC38 cells were co‐injected with either MC38‐derived CTRL or OE cells into BALB‐c/Nude mice (*n* = 5 per group). The representative images of tumors and quantification of tumor weights were shown. (G) Western blot analysis of tumor lysates from the in vivo experiment (F) confirmed the expression of senescence and EMT markers. (H) Representative IHC images of tumor sections from the xenograft models, showing the abundance of m^1^A and expression of p16. Scale bars, 100 µm. (I) TRMT6/61A enhanced cancer stem cell (CSC)‐like properties. Representative bright‐field images and quantification of tumor spheres formed by DLD1 and HCT116 cells co‐cultured with CM derived from CTRL or OE‐WT cells. Scale bars, 50 µm. (J, K) Expression of stemness markers was upregulated in OE‐WT cells. RT‐qPCR analysis revealed increased *NANOG* and *OCT4* mRNA in OE‐WT cells (J), and the Western blot analysis indicated upregulated EpCAM and CD44 protein levels in OE‐WT cells (K). Relative densitometry quantification (normalized to GAPDH) is shown below the bands. Data are presented as mean ± SD from three independent experiments (*n* = 3). ^*^ *p* < 0.05, ^**^ *p* < 0.01, ^***^ *p* < 0.001, ^****^ *p* < 0.0001.

Having established that TRMT6/61A overexpression induces a senescent phenotype (Figure 2), we next sought to investigate the paracrine effects of this SASP. To this end, we collected conditioned medium (CM) from T6‐OE‐WT and the vector group of DLD1 and HCT116 cells to mimic the paracrine signaling environment and co‐cultured it with non‐transfected CRC cells (Figure 5A). We examined the effect on neighboring non‐senescent cancer cells. As hypothesized, CM from T6‐OE‐WT cells significantly enhanced the proliferation, invasion, and migration of non‐transfected CRC cells compared to CM from vector cells (Figure 5B–D). Western blot analysis demonstrated that non‐transfected CRC cells co‐cultured with OE‐WT CM underwent epithelial‐mesenchymal transition (EMT), characterized by upregulation of N‐cadherin, Snail, and Slug (Figure 5E). To confirm the pro‐tumorigenic role of TRMT6/61A‐induced STCs in vivo, we subcutaneously co‐injected non‐transfected CRC cells with either T6‐OE‐WT or vector cells into BALB‐c/Nude mice, respectively. OE‐derived xenografts displayed accelerated tumor growth and increased invasiveness (Figure 5F–H).

Intriguingly, we discovered that senescent tumor cells exhibited senescence‐associated stemness (SAS), a phenomenon highlighting their paradoxical plasticity during senescence [21, 22]. T6‐OE‐WT cells exhibited elevated cancer stem cell (CSC) ‐like properties, including an increase in sphere‐forming ability (Figure 5I) and upregulated expression of stemness markers, including *NANOG*, *OCT4*, *EpCAM*, and *CD44* (Figure 5J,K). These findings suggested that TRMT6/61A‐induced STCs drive CRC progression through SASP‐mediated paracrine activation of EMT, SAS, and proliferative pathways in neighboring tumor cells, which indicates that the crosstalk between STCs and tumor aggressiveness could be exploited as a therapeutic target.

### 2.6 TRMT6/61A‐Induced SASP Facilitates Cancer‐Promoting Macrophage Polarization and CAF Activation

**FIGURE 6:**
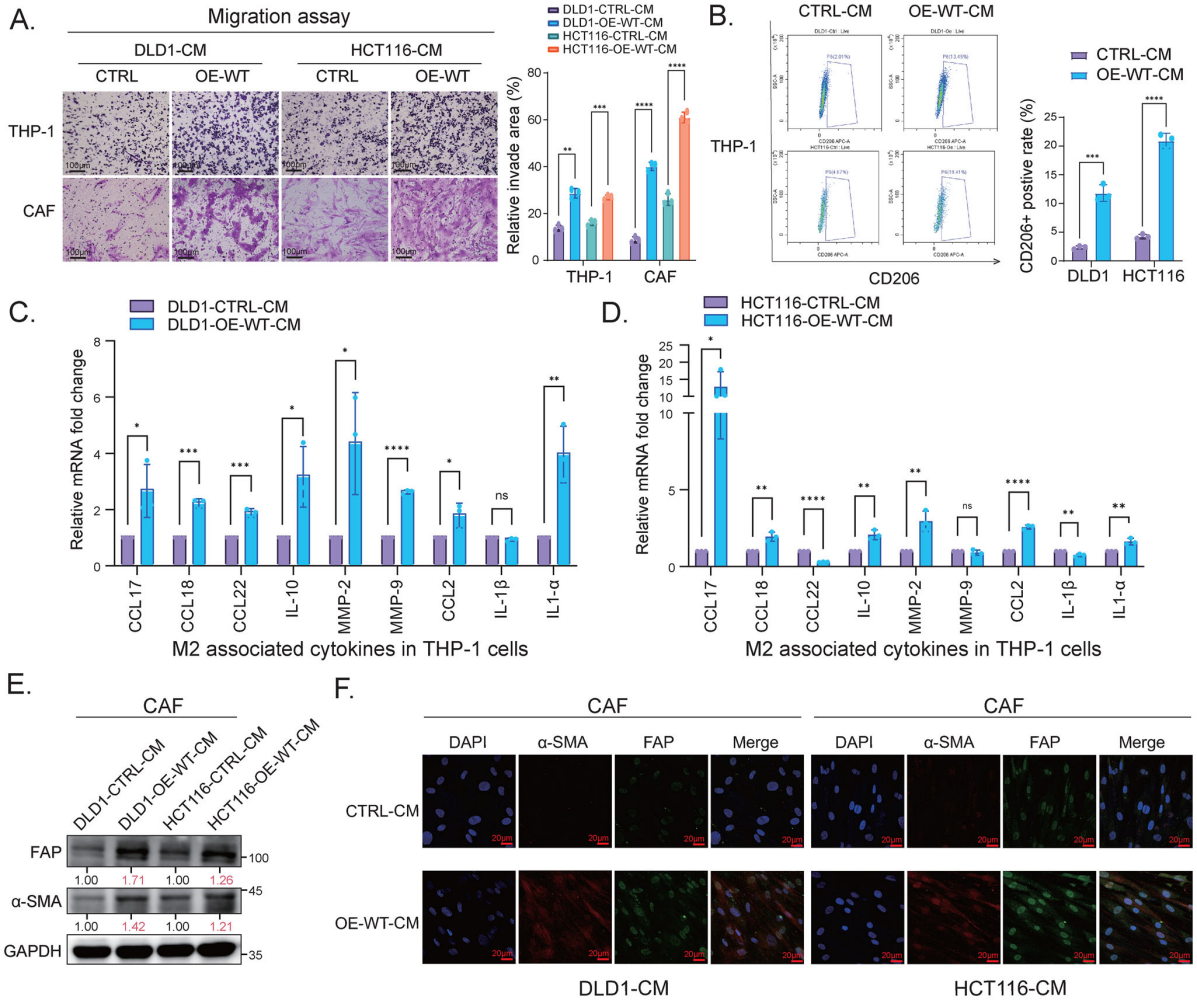
TRMT6/61A‐induced STCs promote M2 macrophage polarization and cancer‐associated fibroblast (CAF) activation. (A) Conditioned medium (CM) derived from the TRMT6/61A‐overexpressing CRC cells (OE‐WT) promoted the migration of THP‐1 monocytes and primary colorectal CAFs compared with that derived from the vector group of CRC cells (CTRL). Representative images (left) and quantification (right) of a transwell migration assay are shown. (B) CM from OE‐WT cells induced the M2‐like polarization of THP‐1 monocytes. Representative flow cytometry plots (left) and quantification (right) showed an increased percentage of CD206‐positive cells after incubation with the indicated OE‐WT‐derived CM. (C, D) RT‐qPCR analysis of M2‐associated cytokine and chemokine expression in THP‐1 monocytes co‐cultured with CM derived from OE‐WT and CTRL groups of DLD1 (C) and HCT116 (D) cells. The expression levels were normalized by the expression in CTRL‐CM‐treated groups. (E) Western blot analysis showing the increased expression of CAF activation markers, including Fibroblast Activation Protein (FAP) and α‐Smooth Muscle Actin (α‐SMA), in CAFs cultured with CM derived from OE‐WT cells. Relative densitometry quantification (normalized to GAPDH) is shown below the bands. Blots are representative of three independent biological replicates (*n* = 3). (F) immunofluorescence assay confirmed the increased expression and co‐localization of FAP (green) and α‐SMA (red) in CAFs upon treatment with OE‐WT‐derived CM. Nuclei were counterstained with DAPI (blue). Scale bars, 20 µm. Data were presented as mean ± SD from three independent biological replicates (*n* = 3). ^*^ *p* < 0.05, ^**^ *p* < 0.01, ^***^ *p* < 0.001, ^****^ *p* < 0.0001.

We next assessed the SASP's impact on non‐tumor components of the TME, as predicted by our single‐cell analysis. We co‐cultured the CM from T6‐OE‐WT and the vector group of CRC cells with THP‐1 monocytes. As a result, the CM from T6‐OE‐WT cells significantly enhanced THP‐1 migration (Figure 6A). In addition, the flow cytometry confirmed increased CD206+ M2 macrophages (Figure 6B), and the expression of M2 macrophage markers and cytokines was elevated in THP‐1 cells exposed to the CM from T6‐OE‐WT cells (Figure 6C,D), suggesting a SASP‐driven immunosuppressive reprogramming. Furthermore, we revealed that the CM from T6‐OE‐WT cells promoted CAF migration (Figure 6A) and activation (Figure 6E,F). Together, these results demonstrate that the SASP production initiated by TRMT6/61A is sufficient to create a pro‐tumorigenic microenvironment. To validate this finding in patients, we examined the association between m^1^A levels and TME remodeling in CRC specimens. Strikingly, quantitative IHC analysis (Figure S1G) revealed a significant positive correlation between the m^1^A score and the M2 macrophage marker CD163 (*r* = 0.64, *p* < 0.001), which was consistent with our in vitro finding that the TRMT6/61A‐m^1^A axis actively drives an immunosuppressive microenvironment in human colorectal cancer.

### 2.7 The Pro‐Tumorigenic Activity of TRMT6/61A‐Induced SASP is Dependent on the NF‐κB Pathway

**FIGURE 7:**
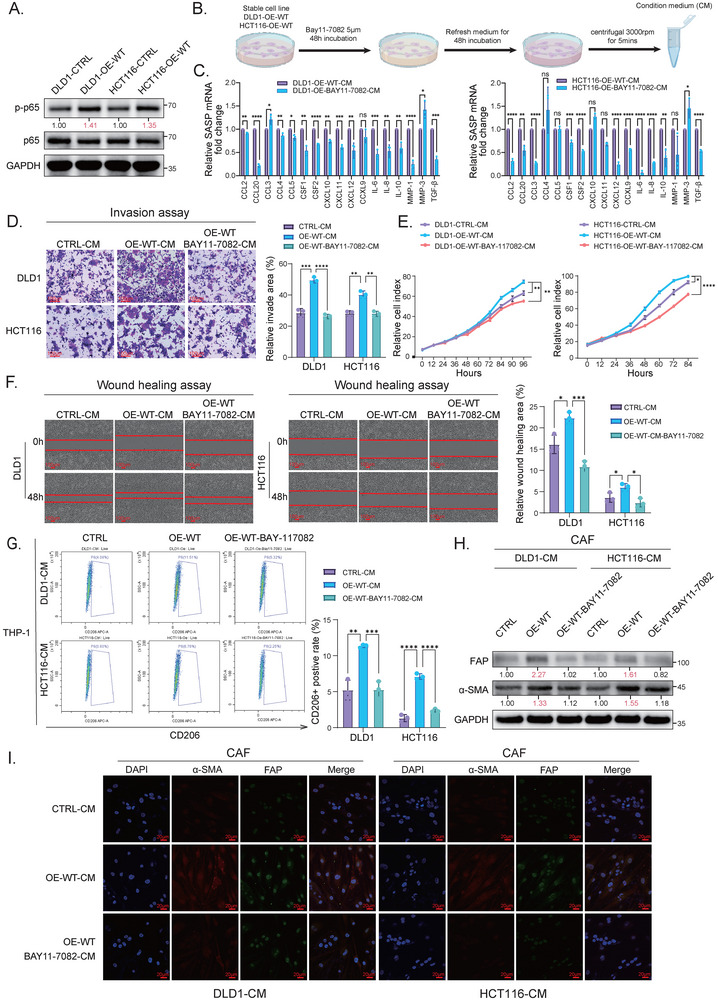
NF‐κB/SASP is essential for pro‐tumorigenic effects of senescent CRC cells (A) Western blot analysis showing an activation of the NF‐κB pathway in TRMT6/61A‐overexpressing (OE‐WT) DLD1 and HCT116 cells compared with vector group (CTRL) cells, as indicated by increased levels of phosphorylated p65 (p‐p65). Relative densitometry quantification (normalized to GAPDH) is shown below the bands. Blots were representative of three independent biological replicates (*n* = 3). (B) Schematic diagram of the experimental design for the NF‐κB inhibition experiments. CM was collected from OE‐WT cells pre‐treated with or without the NF‐κB inhibitor, BAY11‐7082. (C) RT‐qPCR analysis showing that NF‐κB inhibition abrogated the SASP gene signature in OE‐WT cells. (D, E, F) NF‐κB/SASP inhibition in STCs abolished its pro‐malignant effects on recipient cancer cells. Quantification and representative images were shown for the transwell invasion (D), real‐time cell proliferation (E), and wound‐healing (F) assays. The non‐transfected CRC cells were cultured with the CM derived from CTRL cells, OE‐WT cells, or OE‐WT cells pre‐treated with BAY11‐7082. Scale bars for (F), 100 µm. (G) NF‐κB/SASP inhibition rescued the STC‐induced M2 polarization. The flow cytometry showed that the CM from BAY11‐7082‐treated OE‐WT cells did not increase the proportion of CD206‐positive THP‐1 cells. (H,I) NF‐κB /SASP inhibition prevented STC‐induced activation of cancer‐associated fibroblasts (CAFs). Western blot analysis (H) and immunofluorescent assay (I) showed that CM from BAY11‐7082‐treated OE‐WT cells failed to upregulate the activation markers FAP (green) and α‐SMA (red). Relative densitometry quantification (normalized to GAPDH) is shown below the bands in (H). Nuclei were counterstained with DAPI (blue). Scale bars for (I), 20 µm. Data were presented as mean ± SD from three independent biological replicates (*n* = 3). ^*^ *p* < 0.05, ^**^ *p* < 0.01, ^***^ *p* < 0.001, ^****^ *p* < 0.0001.

Our results have established a cascade where the TRMT6/61A‐ARG2 axis activates mTOR signaling to induce senescence (Figure 3), which in turn reshapes the TME via SASP (Figures 5 and 6). Since the NF‐κB pathway is a canonical regulator of SASP production and a key downstream effector of mTOR [23], we hypothesized that NF‐κB is the critical mediator of the pro‐tumorigenic effects of the SASP in our system. To test this, we first confirmed that the NF‐κB pathway was indeed activated in T6‐OE‐WT cells (Figure 7A). To confirm the role of the NF‐κB pathway in TRMT6/61A‐induced STCs SASP, we treated T6‐OE‐WT cells with NF‐κB inhibitor BAY11‐7082 and collected the CM (Figure 7B). This treatment effectively abolished the transcriptional upregulation of multiple SASP factors (Figure 7C), and the resulting CM was used for subsequent functional assays. The CM from BAY11‐7082‐treated T6‐OE‐WT cells failed to enhance the invasion, wound healing ability, and proliferation of non‐transfected CRC cells compared to the CM from untreated T6‐OE‐WT cells (Figure 7D–F). In addition, the immunosuppressive and stromal‐activating effects of SASP were also found to be NF‐κB‐dependent. The CM from BAY11‐7082‐treated T6‐OE‐WT cells reduced M2 polarization of THP‐1 monocytes (Figure 7G) and inhibited CAF activation (Figure 7H,I). These rescue experiments definitively establish that the entire repertoire of pro‐tumorigenic activities orchestrated by TRMT6/61A‐induced STCs is mechanistically dependent on the NF‐κB‐mediated SASP. This completes the mechanistic loop from epitranscriptomic modification to TME reprogramming.

## 3 Discussion

In this study, we elucidate a novel epitranscriptomic mechanism driving colorectal cancer (CRC) progression, beginning with our observation that RNA m^1^A methylation is aberrantly elevated in CRC tissues and correlates with poor patient prognosis. We establish that this is driven by the methyltransferase complex TRMT6/61A, which induces tumor cell senescence in a manner dependent on its catalytic m^1^A writer activity. Mechanistically, we demonstrate that TRMT6/61A‐mediated tRNA‐m^1^A modification selectively enhances the translational efficiency of *ARG2*, subsequently activating the mTOR and NF‐κB signaling pathways to establish the senescent phenotype and promote the production of a pro‐tumorigenic Senescence‐Associated Secretory Phenotype (SASP). Functionally, this STC‐derived SASP reshapes the tumor microenvironment by promoting the proliferation, invasion, and stemness of neighboring non‐senescent cancer cells, while also driving the polarization of immunosuppressive M2 macrophages and the activation of CAFs. Collectively, our findings uncover the TRMT6/61A‐ARG2 axis as a critical regulator of pro‐tumorigenic senescence in CRC, providing a strong rationale for targeting this pathway as a novel senomorphic therapeutic strategy.

Our identification of the TRMT6/61A complex as a central driver of CRC progression is powerfully corroborated by the concurrent findings of Tao et al. [24]. Yet, a compelling dichotomy emerges: while Tao et al. describe a cell‐intrinsic mechanism fueling histone‐dependent proliferation, our study uncovers a distinct, non‐cell‐autonomous pathway driving senescence. We propose that these phenotypes are not contradictory but represent a ‘dual‐function’ oncogenic strategy. Internally, *TRMT6* supports the rapid cycling of the proliferative tumor bulk; concurrently, it pushes a subset of cells into a SAS state [21]. These arrested cells function as metabolic ‘secretory hubs’, utilizing the SASP to remodel the microenvironment and promote malignancy in trans. This suggests that CRC employs an evolutionary ‘bet‐hedging’ strategy, leveraging epitranscriptomic regulation to balance expansive growth with environmental adaptation [25, 26]. Consequently, this unified model offers a far more comprehensive explanation for the robust correlation between TRMT6 overexpression and poor patient prognosis than either mechanism alone, underscoring the complex adaptive value of m^1^A modification in tumorigenesis.

Translational regulation, significantly influenced by tRNA modifications such as m^1^A, is crucial in cancer biology [27, 28, 29]. Unlike some models suggesting m^1^A58 boosts global translation initiation via tRNA^iMet^ [20, 30], we found no such elevation in CRC cells. Instead, TRMT6/61A overexpression led to specific tRNA‐Glu‐CTC hypermethylation, consistent with findings in hepatocellular carcinoma [15], suggesting that cancer cells repurpose m^1^A for pro‐oncogenic programs by modulating the elongation‐phase dynamics of specific codons. Structural function of TRMT6/61A in CRC was strictly dependent on its catalytic m^1^A methyltransferase activity—mutations at critical sites abolished m^1^A deposition and senescence induction [31]. This contrasts with reports of non‐catalytic TRMT6/61A functions in hematopoietic aging [13], highlighting differing dependencies in physiological versus pathological contexts. Tumors appear to co‐opt tRNA modifications; the enhanced translation of GAG codon‐enriched mRNAs via tRNA^Glu‐CTC^ m^1^A hypermethylation may reflect the metabolic demands of CRC [32]. This strict catalytic dependence for senescence induction suggests targeting TRMT6/61A's enzymatic site as a potential therapeutic strategy for CRC.

ARG2, a mitochondrial enzyme that hydrolyzes arginine [33], is implicated in tumor progression; however, its role in STCs was unclear. While ARG2 can induce senescence in non‐malignant cells via mTOR pathway activation [19, 34], our research found that TRMT6/61A‐mediated m^1^A methylation specifically upregulates *ARG2* mRNA translation efficiency. This is achieved by enhancing the decoding of specific codons, such as those for glutamic acid (Glu) and aspartic acid (Asp), thereby promoting ARG2 protein synthesis. This increase in ARG2 activated the mTOR signaling pathway, inducing tumor cell senescence, a phenotype reversed by mTOR inhibition with rapamycin. Our discovery of the TRMT6/61A‐ARG2 axis driving a senescence‐associated pro‐tumorigenic program is particularly significant. It reveals a novel layer of regulation for this complex, distinct from its recently described role in directly fueling cell‐intrinsic proliferation through the translational control of cell‐cycle‐related proteins like histones. This highlights the multifaceted role of tRNA m^1^A modification in CRC, which can not only promote cancer growth directly [24], but also orchestrate a tumor‐promoting microenvironment through SASP.

Our data demonstrate that TRMT6/61A‐driven cells acquire a unique phenotype characterized by stable growth arrest concomitant with the upregulation of key stemness factors, including *NANOG*, *OCT4*, and *EpCAM*. This indicates that high TRMT6/61A expression reprograms tumor cells into a malignant SAS state, wherein they leverage the senescent program to secrete a potent SASP that remodels the microenvironment, while simultaneously utilizing acquired stemness traits to drive invasiveness and therapeutic resistance [35, 36, 37]. Consequently, rather than functioning as a tumor‐suppressive barrier, this specific form of senescence fuels tumor progression through non‐cell‐autonomous mechanisms, consistent with the adverse clinical outcomes observed in patients [6, 18].

The pro‐tumorigenic nature of the SASP lies in its complex composition [4], where diverse factors synergize to remodel the TME [38]. Our findings that TRMT6/61A‐induced STCs orchestrate a malignant microenvironment align with this concept, and the specific factors we identified offer mechanistic insight into this process. For example, elevated TGF‐β, as seen in our model, is a canonical activator of CAFs through the SMAD pathway [39, 40], inducing the α‐SMA expression we observed (Figure 6E,F). Simultaneously, the myeloid compartment is subjected to a sophisticated, multistep reprogramming. First, the highly expressed chemokine CCL2 actively recruits monocytes into the tumor microenvironment via CCR2 signaling [41, 42]. Once present, these recruited cells are then educated by other SASP components, such as IL‐6, which drives their polarization toward an immunosuppressive M2 phenotype via the JAK/STAT3 pathway (Figure 6B–D) [43, 44]. The convergence of these distinct yet complementary pathways explains the robust pro‐tumorigenic phenotype and underscores why targeting the upstream master regulator, the NF‐κB pathway, is a more effective strategy than neutralizing any single cytokine.

While arginase inhibitors are currently explored for restoring antitumor immunity [45, 46], our findings suggest a novel approach for both senomorphic and senolytic regimens in CRC. Targeted inhibition of the TRMT6/61A‐ARG2‐NF‐κB axis would act as a functional senomorphic strategy [47]. As demonstrated by our data, this approach effectively neutralizes the pathological SASP and blocks pro‐tumorigenic TME crosstalk. In addition, selective elimination of senescent tumor cells with senolytic drugs or antibody‐drug conjugates, particularly in the CRC patients with a high burden of TRMT6/61A‐ARG2‐dependent senescence, would act as a promising regimen for CRC.

While this study establishes TRMT6/61A‐mediated tRNA‐m^1^A modification as a critical driver of SASP and TME remodeling in CRC, several limitations warrant consideration. First, our work did not delineate the individual contributions of specific SASP components (e.g., IL‐6, TGF‐β, or CCL2) to distinct TME cell populations, such as neighboring tumor cells, CAFs, or macrophages. This limitation stems from the inherent complexity of SASP, which acts synergistically or antagonistically on various cellular targets [23]. Isolating the effects of single SASP factors may oversimplify their biological roles, as their combinatorial actions likely drive context‐dependent outcomes. On the other hand, our focus on TRMT6/61A as the primary m^1^A “writer” overlooks potential contributions from other tRNA methyltransferases. TRMT10C and TRMT61B are known to mediate m^1^A modifications at distinct tRNA positions, and their roles in senescence regulation remain unexplored [48]. Whether these enzymes compensate for or oppose TRMT6/61A activity in STCs and how their dysregulation impacts SASP heterogeneity represents a critical avenue for future investigation.

In summary, our study provides novel insights into the occurrence of STCs and their impact on tumor progression from an epigenetic perspective. Our findings demonstrate that the TRMT6/61A complex‐mediated m^1^A methylation modification promotes the synthesis of ARG2 protein, activates the mTOR signaling pathway, induces the occurrence of STCs, and promotes tumor progression through the SASP. Our results elucidate one of the sources of STCs and offer targeting tRNA‐m^1^A modification as a novel strategy to counteract senescence‐associated immune evasion, complementing existing TIS‐targeted therapies like senolytics.

## 4 Experimental Section

### 4.1 Patient and Sample Collection

A cohort of 58 patients diagnosed with CRC, who underwent surgical resection from January 2012 to December 2015, was identified from the National Basic Research Program of Evolution from Precancerous Disease to Cancer in China (NEPDC cohort, No. 2015CB554000) [49, 50, 51], and the clinicopathological characteristics of these patients were detailed in Table S1. Patients were included if they had histopathologically confirmed CRC, no prior chemotherapy or radiotherapy, and the availability of complete clinical data. Exclusion criteria included a history of other malignancies, inflammatory bowel disease, or inadequate sample quality. Samples were divided into two portions: one portion was snap‐frozen in liquid nitrogen and stored at −80°C for subsequent RNA and protein analyses, while the other was fixed in 10% neutral‐buffered formalin for 24 h and then embedded in paraffin for histopathological examination.

This study was conducted in accordance with the ethical standards of the institutional research committee and the 1964 Declaration of Helsinki and its subsequent amendments. Approval was obtained from the Institutional Review Board (IRB) of the Sixth Affiliated Hospital of Sun Yat‐sen University (No. 2024ZSLYEC‐318). Written informed consent was obtained from all participants before their inclusion in the study.

### 4.2 Cell Culture

Human colorectal cancer cell lines DLD1 (RRID: CVCL_0248) and HCT116 (RRID: CVCL_0291) were obtained from the American Type Culture Collection (ATCC, Manassas, VA, USA) in October 2022. The cell lines were authenticated by short tandem repeat (STR) profiling upon receipt and were routinely tested to be free of mycoplasma contamination. Cells were used within 15 passages from thawing. Cells were cultured in Corning RPMI 1640 medium (Corning, 10‐040‐CVRC) supplemented with 10% Lifeman FBS (Lifeman, FBS001) and 1% ThermoFisher penicillin‐streptomycin (ThermoFisher, 15140122) at 37°C in a humidified atmosphere containing 5% CO_2_. The DNA sequences of TRMT6 (NC_000020.11), TRMT61A (NC_000014.9), ARG2 (NC_000014.9), TRMT6‐Mut, TRMT61A‐Mut, and ARG2‐Mut were cloned into the pCDH plasmid (IGE, China) to construct stably expressed cells with lentiviral vectors.

### 4.3 RNA Extraction and Quantitative Real‐Time PCR

Total RNA was extracted from cells and tissues using the RNA Quick Extraction Kit (GOONIE, 400‐100‐100T) per the manufacturer's instructions. For cDNA synthesis, 1 µg of total RNA was reverse‐transcribed using the HRbio III 1st Strand cDNA Synthesis SuperMix (HRbio, HRF0182) following the manufacturer's instructions. Quantitative real‐time PCR (RT‐qPCR) was performed using the HRbio SYBR Green Master Mix (HRbio, HRF0042) on the QuantStudio 7 Real‐Time PCR System (Applied Biosystems). Specific cycling parameters, data acquisition, and analysis methods were consistent with our previous publication [52]. GAPDH served as the internal control for normalization. Primer sequences for the detected genes were listed in Table S2.

### 4.4 m^1^A Dot Blot

Dot blot analysis was used to assess the global m^1^A modification levels in total RNA. Briefly, the 1000, 500, and 250 ng of total RNA were denatured by heating at 65°C for 5 min and immediately cooled on ice. The denatured RNA was then spotted onto a positively charged nylon membrane (Solarbio, YA1760) and air‐dried. The membrane was cross‐linked using UV irradiation at 120 mJ/cm^2^ for 30 min. The subsequent immunodetection steps, including membrane blocking, antibody incubations, washing, and chemiluminescent detection, were mainly performed according to our previously published protocol [49]. Specifically for this study, the membrane was blocked with 5% non‐fat dry milk (prepared in 1× TBST) for 1 h at room temperature, followed by incubation with a primary anti‐m^1^A antibody (MBL, D345‐3) overnight at 4°C. After washing three times with TBST, the membrane was incubated with horseradish peroxidase (HRP)‐conjugated secondary antibody (Proteintech, SA00001‐1‐500ul) for 1 h at room temperature. Detection was performed using an enhanced chemiluminescence (ECL) reagent (Meilunbio, MA0186‐2) and visualized on an imaging system (Azure, USA).

### 4.5 RNA‐Seq

RNA‐seq analysis was performed on DLD1 cells with overexpression of *TRMT6* and *TRMT61A*, using three biological replicates for each group. Total RNA was processed by CloudSeq Biotech Inc. (Shanghai, China). Ribosomal RNA was removed using the GenSeq rRNA Removal Kit (GenSeq, Inc.), and libraries were constructed using the GenSeq Low Input RNA Library Prep Kit (GenSeq, Inc.), following the manufacturer's protocol. The libraries were evaluated for quality using the BioAnalyzer 2100 system (Agilent Technologies, Inc., USA) and sequenced on an Illumina platform with 150‐bp paired‐end reads.

### 4.6 RNA‐Seq Data Processing and Analysis

Sequencer‐derived paired‐end reads underwent quality control with a Q30 threshold. Low‐quality reads and 3' adapters were removed using Cutadapt (v1.9.3). Clean reads were aligned to the human reference genome (GRCh38) using HISAT2 (v2.0.4). Gene‐level counts were calculated with HTSeq (v0.9.1), and differentially expressed mRNAs were identified using edgeR, with thresholds set at *p* < 0.05 and |log2(fold change)| > 1. GO and KEGG pathway enrichment analyses were conducted on the differentially expressed mRNAs.

### 4.7 m^1^A‐tRNA‐Seq

CloudSeq Inc. (Shanghai, China) conducted the tRNA sequencing service. Total RNA was size‐selected for small RNA fractions (<200 nt) using the MirVana Isolation Kit (ThermoFisher, USA). The enriched small RNAs were de‐aminoacylated in 0.1 m Tris–HCl (pH 9.0) and 1 mm EDTA for 30 min at 37°C. Libraries were constructed using the GenSeq Small RNA Library Prep Kit (GenSeq, Inc.) according to the manufacturer's protocol. After selecting the library size for tRNA fractions, sequencing was performed on the NovaSeq platform (Illumina, USA).

### 4.8 tRNA m^1^A‐Seq Data Processing and Analysis

To prepare the tRNA reference library, tRNA sequences were adapted from the tRNAScan‐SE library by appending CCA to the tRNAs obtained from the Genomic tRNA Database (http://gtrnadb.ucsc.edu/GtRNAdb2/genomes/). Isoformers with identical scores were consolidated to simplify identity assignment and reduce redundancy in reference genes and pseudogenes. Raw reads generated from the sequencer underwent quality control using a Q30 threshold, and adapter sequences were trimmed using Cutadapt (version 1.9.3). Reads with a length of ≥15 nucleotides were retained. Trimmed reads were aligned to the custom tRNA library using Bowtie2 with sensitive options. Mapped reads for each tRNA were quantified using Samtools, and differential expression of tRNA between experimental conditions was analyzed using EdgeR.

### 4.9 Ribo‐Seq

Ribosome profiling sequencing (Ribo‐Seq) was performed to examine genome‐wide RNA translation. Ribosome‐nascent peptide complexes were treated with low concentrations of RNase to degrade unprotected RNA, isolating ribosome‐protected fragments (∼30 nucleotides). Libraries were prepared using the NEBNext Ultra RNA Library Prep Kit and sequenced on an Illumina NovaSeq 6000 platform, generating paired‐end reads of 150 bp.

### 4.10 Ribo‐Seq Data Processing and Analysis

Adapter sequences were trimmed using the Cutadapt software, and reads of 25–35 bp were retained. Bowtie2 was used to remove reads aligning to rRNA and tRNA, and the remaining reads were mapped to the Ensembl reference genome (Version 91) and transcriptome using HISAT2 and Bowtie2. Ribosome trinucleotide periodicity and codon usage were analyzed using the riboWaltz package. Open reading frames (ORFs) were identified using Price software, and read counts were calculated with feature counts.

Translation efficiencies were determined by comparing ribosome profiling and RNA‐seq data, and differences were identified using the DESeq2 package. Genes with fold changes >2 and *p* < 0.05 were considered significant. GO and KEGG enrichment analyses were performed with clusterProfiler in R, and heatmaps were created using heatmap. All RPKM values were computed using DESeq2's FPKM function.

### 4.11 Bioinformatic Analysis of Public Datasets

To validate the prognostic significance of m^1^A writers, survival analyses were conducted using an integrated pan‐cancer database. Kaplan‐Meier survival curves for CRC cohorts were generated via the Kaplan‐Meier plotter online tool [53], which utilizes gene expression and clinical data from multiple public sources, including gene expression omnibus (GEO) and the cancer genome atlas (TCGA).

The single‐cell RNA sequencing data from CRC samples were obtained from GEO with the identifier GSE196964. The data processing was performed using the Seurat package (v5.3.0), which involved standard quality control, filtering, normalization, identification of highly variable genes, scaling, and principal component analysis (PCA). Significant principal components were then utilized for uniform manifold approximation and projection (UMAP) for visualization and graph‐based clustering. Cell clusters were annotated into distinct cell types based on the expression of canonical marker genes, as identified in our previously published article [54]. The relative proportions of these identified cell types within samples were also determined, and marker gene expression across cell types was characterized.

Key downstream analyses were conducted to investigate TME reprogramming associated with senescent tumor cells. Differentially expressed gene (DEG) analysis was performed between specified cell lineages to identify altered gene expression profiles, with specific annotation of SASP genes derived from previously published literature [23]. Intercellular communication networks were inferred using CellChat (v2.1.2), including sub‐classification of tumor cells based on ARG2 expression. This approach was used to explore ligand‐receptor interactions and to assess the aggregated outgoing and incoming signaling strength of key SASP‐related signaling pathways across different cell populations within the TME.

### 4.12 Protein Isolation and Western Blotting

Protein extraction from cells, including lysis in RIPA buffer (Thermo Fisher, #89900) with protease and phosphatase inhibitors (Roche, #04906837001 and #04693159001), sonication, clarification, and BCA protein quantification (Thermo Scientific, #23227), was performed as previously detailed [55, 56].

A total of 30 µg of protein was resolved on SDS‐PAGE gels and transferred to PVDF membranes. Membranes were blocked with 5% non‐fat milk in TBST for 1 h at room temperature, followed by overnight incubation at 4°C with primary antibodies. After washing, the membranes were incubated with HRP‐conjugated secondary antibodies for 1 h at room temperature. Detection was performed using an ECL reagent (Meilunbio, MA0186‐2) and visualized on an imaging system (Azure, USA).

### 4.13 SA‐β‐Gal Staining

Cellular senescence was assessed by senescence‐associated β‐galactosidase (SA‐β‐Gal) activity, a standard marker for senescent cells [57]. For this study, the Senescence β‐Galactosidase Staining Kit (Cell Signaling Technology, #9860) was used according to the manufacturer's protocol. Briefly, cells were seeded into 12‐well plates and cultured until 80–90% confluence. After washing with PBS, cells were fixed in the provided fixative solution for 15 min at room temperature. The cells were then incubated overnight at 37°C in a staining solution containing X‐gal substrate, with the incubation chamber maintained at atmospheric pressure. Senescent cells were identified by blue staining under a light microscope, and the percentage of SA‐β‐Gal‐positive cells was calculated from five random fields per well. The percentage of SA‐β‐Gal‐positive cells was determined by quantifying both positive (blue‐stained) and total cells from three randomly selected high‐power fields (100×) per well, using the cell counter plugin of ImageJ software. Data were presented as mean ± SD from three independent experiments.

### 4.14 Dual‐Luciferase Reporter System

The dual‐luciferase reporter assay was performed using the Dual‐Luciferase Reporter Assay System (Promega, USA) according to the manufacturer's instructions. Cells were co‐transfected with the firefly luciferase reporter plasmid (F‐luc) and Renilla luciferase plasmid (R‐luc) as an internal control using Lipofectamine 3000 (Thermo Fisher Scientific, USA). We constructed four plasmids with six‐repeated UUG, GAG, GCA, and GAC inserted before the F‐luc, respectively. After 24–48 h of transfection, cells were lysed using the provided lysis buffer. The luciferase activities of firefly and Renilla were measured sequentially using a luminometer. The relative luciferase activity was calculated as the ratio of firefly to Renilla luciferase activity to normalize for transfection efficiency.

### 4.15 Wound‐Healing Assay

A wound‐healing assay was performed to evaluate cell migration. DLD1 and HCT116 cells were seeded in 12‐well plates and cultured until a monolayer was formed. A sterile 200 µL pipette tip created a straight scratch across the cell monolayer. The detached cells were removed by washing with PBS and cultured in a serum‐free medium. Images of the wound area were captured every 2–4 h using the Incucyte Zoom system (Essen BioSciences, USA). The wound closure was quantified by measuring the remaining wound area using ImageJ software.

### 4.16 Transwell Assay

A Transwell assay was conducted to evaluate the migratory and invasive capabilities of DLD1 and HCT116 cells. For migration assays, 1×10^5^ cells suspended in 100 µL medium containing 1% FBS were seeded in the upper chamber of a 24‐well Transwell insert. The lower chamber was filled with 700 µL of medium containing 10% FBS or conditioned medium (CM) as a chemoattractant. After 48 h of incubation, cells were fixed with 4% paraformaldehyde and stained with 0.1% crystal violet. Images were captured under a light microscope. For the invasion assay, the upper compartment of the chamber was precoated with Matrigel (Corning, MA, USA), and the subsequent steps were the same as those for the migration assay.

### 4.17 Flow Cytometry

Monoclonal antibodies against CD206 (BioLegend, 321110) were purchased from BioLegend. The cells were stained with antibodies (1:200) at 4°C for 30 min. For intracellular staining, after surface antibody staining, cells were fixed and permeabilized using a Fixation/Permeabilization Solution Kit (BD Biosciences, USA) as previously described [58], followed by staining with antibodies against intracellular molecules. Data were collected with a flow cytometer (CytoFLEX S, USA) and analyzed in FlowJo.

### 4.18 Tumor Xenograft Experiments

All animal experiments were approved by the Animal Care Committee of the Sixth Affiliated Hospital of Sun Yat‐sen University (No. IACUC‐2024051101). BALB‐C/Nude mice (4–5 weeks old, 18–20 g) were obtained from GemPharmatech Co., Ltd (Guangdong, China).

For the in situ tumor formation model, 1 × 10^6^ MC38 stable cell lines were resuspended in 50 µL of PBS with equal wild‐type MC38 and injected into the cecum of BALB‐C/Nude mice. Mice were sacrificed 28 days after implantation, and tumor specimens were collected for weight measurement and subsequent experiments.

### 4.19 Hematoxylin and Eosin (H&E) and Immunohistochemistry (IHC) Staining

All tissues were embedded in paraffin wax and sectioned into 3 µm slices. Standard protocols of IHC have been described previously [49]. The deparaffinized sections were incubated with anti‐m^1^A (MBL, D34‐5‐3), anti‐p21 (Cell Signaling Technology, 9449), and anti‐p16 (Proteintech, 10883‐1‐AP). IHC scoring was performed by two pathologists blinded to the clinical data, using the standard Immunoreactive Score (IRS) method [59].

### 4.20 Immunofluorescence Staining (IF)

Cells were cultured in 35‐mm glass‐bottom dishes. Immunofluorescence staining was performed mainly according to our previously described methods [49]. Briefly, all cells were cultured in 35‐mm glass‐bottom dishes. The cells were fixed with 4% paraformaldehyde and then made permeable using Triton X‐100. Following this, the cells were blocked with 5% bovine serum albumin and then subjected to incubation with primary antibodies targeting α‐SMA and FAP. The sections or glass‐bottom dishes were then incubated with Alexa Fluor 488‐ or 594‐conjugated secondary antibodies. The nuclei were counterstained with DAPI. Visualization of the dyed cells was achieved using a fluorescent microscope.

### 4.21 Statistical Analysis

Statistical analyses were conducted using GraphPad Prism 8.0, SPSS 25.0, and R (version 4.3.1). Data normality was assessed using the Shapiro‐Wilk test, and homogeneity of variance was evaluated using Levene's test. For comparisons between two groups, a two‐tailed Student's *t*‐test was used for normally distributed data, while the non‐parametric Wilcoxon rank‐sum test or Wilcoxon signed‐rank test (for paired samples) was applied for non‐normal data. For comparisons among multiple groups, one‐way or two‐way analysis of variance (ANOVA) followed by Tukey's post‐hoc test was performed; non‐parametric data were analyzed using the Kruskal‐Wallis test. Pearson's correlation analysis was performed to determine the correlation between two continuous variables. Survival analysis was performed using Kaplan‐Meier curves, and significance was assessed using the log‐rank test. Categorical data were analyzed using Chi‐square tests or Fisher's exact test. Data were presented as mean ± standard deviation (SD) unless otherwise stated. All experiments were repeated at least three times. A *p*‐value of < 0.05 was considered statistically significant.

### 4.22 Ethics Approval Statement

This study has been approved by the Institutional Review Board of the Sixth Affiliated Hospital of Sun Yat‐sen University (Guangzhou, China).

## Author Contributions

T.‐Y.L., M.‐Z.H. and J.‐L.C. contributed equally to this work. Y.‐X.L., M.‐J.H., J.‐X.L., and H.‐C.Y. conceived the research and supervised the study. T.‐Y.L., M.‐Z.H., and J.‐L.C. performed the investigation, developed the methodology, and wrote, reviewed, and edited the manuscript. X.L., Y.‐K.X., R.‐Z.H., Z.‐X.W., Y.‐GG., Z.‐M.L., J.‐R.W., Y.‐M.X., and Y.‐H.W. contributed to data acquisition. T.‐Y.L., M.‐Z.H., X.‐X.L., and X.‐L.W.W. analyzed and interpreted the data. All authors approved the final publication

## Patient Consent Statement

Written informed consent was obtained from all participants included in the study.

## Conflicts of Interest

The authors declare no conflicts of interest.

## Supporting information




**Supporting File 1**: advs73426‐sup‐0001‐SuppMat.docx

## Data Availability

The RNA‐seq, m^1^A‐tRNA‐seq, and Ribo‐seq data generated in this study have been deposited in the gene expression omnibus (GEO) database under accession numbers GSE290123 and GSE290126. The publicly available single‐cell RNA sequencing dataset analyzed in this study is available in the GEO database under accession number GSE196964. The clinical data from the NEPDC cohort contain sensitive patient information and are therefore not publicly available due to privacy and ethical restrictions, but are available from the corresponding author upon reasonable request and subject to institutional review board approval. All other data supporting the findings of this study are available within the article and its Supporting Information files or from the corresponding author upon reasonable request.
