# Supplementary material for: m1A‐Dependent TRMT6/61A‐ARG2 Axis Drives Protumorigenic Senescence by Remodeling the Tumor Microenvironment
Source: Adv Sci (Weinh). 2026 Jan 8;13(13):e18536. doi: 10.1002/advs.202518536 (PMC12955887; doi:10.1002/advs.202518536)
Supplement: Supplementary file 1 — Supporting File 1: advs73426‐sup‐0001‐SuppMat.docx [file ADVS-13-e18536-s001.docx]

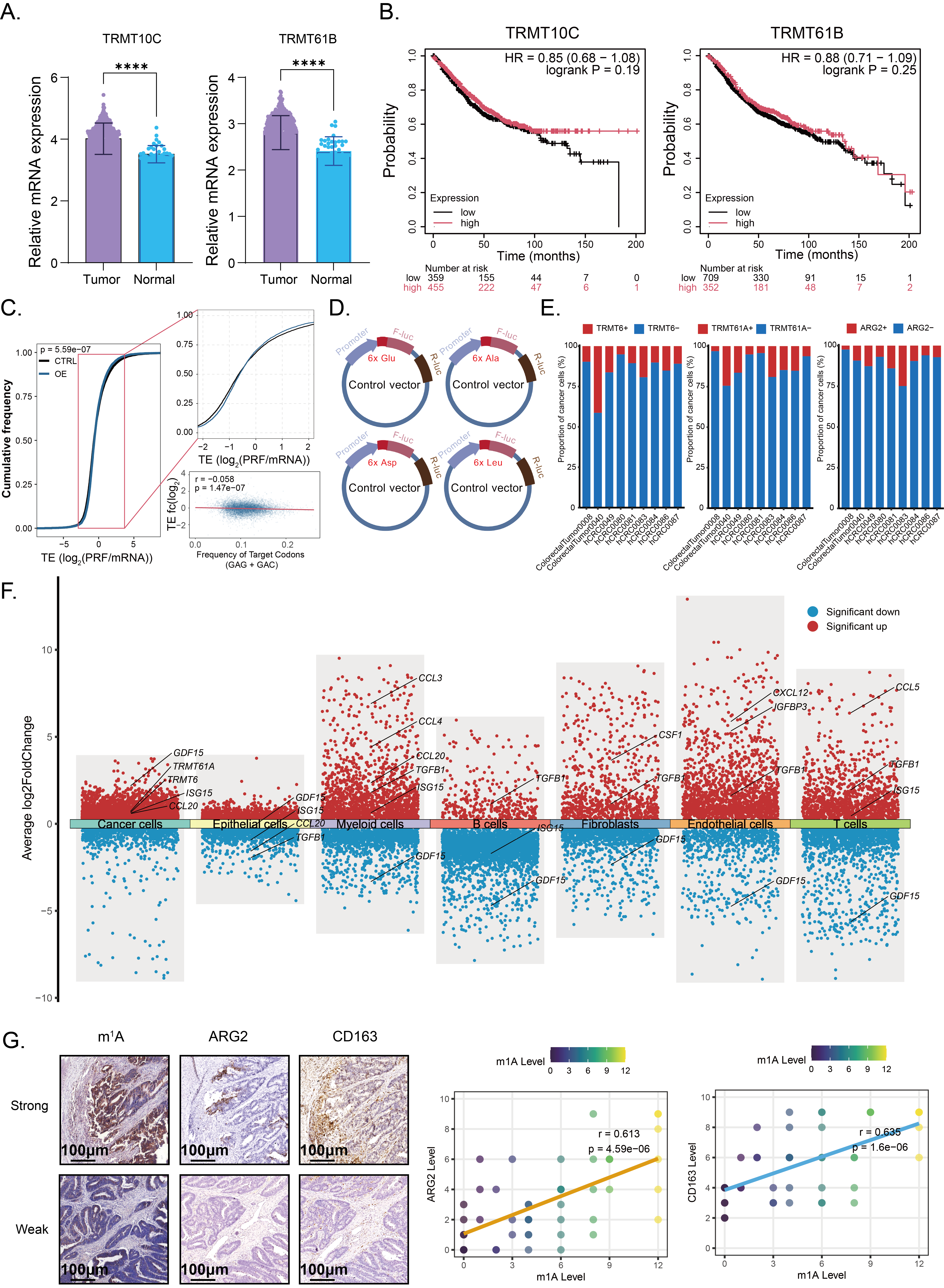


**Figure S1. Distribution of m^1^A modification “writer” and overall survival in an external validation cohort.**

(A) The expression distribution of TRMT10C and TRMT61B of m^1^A modification between paired normal (blue) and CRC (red) tissues using public CRC datasets. (B) Kaplan–Meier plots of overall survival based on TRMT10C and TRMT61B of m^1^A expression levels in the kmplot database. (C) Global analysis of translational efficiency (TE). Left: Cumulative frequency distribution of TE in CTRL and OE-WT cells (p = 5.59e-07). Right: Scatter plot showing the correlation between the TE fold change (OE-WT/CTRL) and the frequency of m^1^A -target codons (GAG and GAC) in transcripts. The Pearson correlation coefficient (r = -0.058) indicates no simple linear correlation, supporting a specific rather than global translational enhancement. (D) Schematic diagram of the dual-luciferase reporter constructs. Reporter plasmids were constructed by inserting six-repeated cognate codons for Glu (GAG), Asp (GAC), Ala (GCA), or Leu (UUG) immediately upstream of the firefly luciferase (F-luc) gene to assess codon-specific translational regulation. (E) Stacked bar charts quantifying the intratumoral heterogeneity of target gene expression. The plots illustrate the proportion of cancer cells expressing TRMT6, TRMT61A, and ARG2 versus non-expressing cells within individual CRC patient samples from the single-cell cohort. (F) Differential expression analysis of major cell types in the CRC single-cell RNA-seq dataset. Scatter plots display the average log2 fold change of genes, with significantly upregulated genes shown in red and downregulated genes in blue. Key genes, including m^1^A writers (TRMT6, TRMT61A) and SASP factors (e.g., GDF15, CCL20, TGFB1, IGFBP3, CXCL12, CCL3), are highlighted, particularly within the cancer cell and stromal cell clusters. (G) Left: Representative IHC images of serial sections from the NEPDC cohort (n = 58), showing the expression of m^1^A, ARG2, and the M2 macrophage marker CD163. Representative regions with strong and weak staining are shown. Scale bars, 100 μm. Right: Association analysis of m^1^A abundance with the expression of ARG2 and CD163 based on their IHC scores in CRC tumor tissues (NEPDC cohort, n = 58). Pearson’s correlation coefficient (r) and p-value were indicated. All data are presented as mean ± SD. ****p < 0.0001.

**Table S1. Baseline characteristics**

|  | | m1A Level | |  |
| --- | --- | --- | --- | --- |
| Variable | Overall  n = 58^a)^ | High  n = 29 ^a)^ | Low  n = 29 ^a)^ | p-value ^b)^ |
| Age at admission | 60.5 (52.5, 70.5) | 58.5 (49.0, 70.5) | 60.5 (54.0, 70.5) | 0.5 |
| Gender |  |  |  | 0.5 |
| Missing | 10 (17%) | 5 (17%) | 5 (17%) |  |
| Male | 28 (48%) | 16 (55%) | 12 (41%) |  |
| Female | 20 (34%) | 8 (28%) | 12 (41%) |  |
| Tumor length | 4.0 (2.9, 5.0) | 4.5 (3.6, 5.8) | 3.7 (2.5, 4.3) | 0.032 |
| Differentiation degree |  |  |  | 0.2 |
| Missing | 6 (10%) | 3 (10%) | 3 (10%) |  |
| Low differentiation | 4 (6.9%) | 2 (6.9%) | 2 (6.9%) |  |
| High differentiation | 19 (33%) | 6 (21%) | 13 (45%) |  |
| moderate differentiation | 29 (50%) | 18 (62%) | 11 (38%) |  |
| T stage |  |  |  | 0.4 |
| T1 | 5 (8.6%) | 1 (3.4%) | 4 (14%) |  |
| T2 | 6 (10%) | 2 (6.9%) | 4 (14%) |  |
| T3 | 44 (76%) | 25 (86%) | 19 (66%) |  |
| T4 | 3 (5.2%) | 1 (3.4%) | 2 (6.9%) |  |
| N stage |  |  |  | 0.2 |
| N0 | 43 (74%) | 22 (76%) | 21 (72%) |  |
| N1 | 10 (17%) | 3 (10%) | 7 (24%) |  |
| N2 | 5 (8.6%) | 4 (14%) | 1 (3.4%) |  |
| M stage |  |  |  | >0.9 |
| M0 | 57 (98%) | 28 (97%) | 29 (100%) |  |
| M1 | 1 (1.7%) | 1 (3.4%) | 0 (0%) |  |
| ^b)^Median (Q1, Q3); n (%) | | | | |
| ^b)^Wilcoxon rank sum test; Pearson's Chi-squared test; Fisher's exact test | | | | |

**Table S2. RT-qPCR primers used in this study**

| Gene | Forward sequences (5’-3’) | Reverse sequences (5’-3’) |
| --- | --- | --- |
| TRMT6 | GGTGCTGAAACGTGAAGATGT | CTTGGGCTGTAGACTTCCTCC |
| TRMT61A | TGGCTAAAGAGCATTCTGCTAAG | TGTGTAGTCGAAGCATGTTGTG |
| CCL2 | AgCAgCAAgTgTCCCAAAgA | TTgggTTTgCTTgTCCAggT |
| CCL3 | AgTTCTCTgCATCACTTgCTg | CggCTTCgCTTggTTAggAA |
| CCL4 | gCTgCTCAgAgACAggAAgT | ACAggAACTgCggAgAggAg |
| CCL5 | TCCCACAggTACCATgAAggTC | gCAATgTAggCAAAgCAgCAg |
| CCL17 | ATggCCCCACTgAAgATgCTT | TgAACACCAACggTggAgg |
| CCL18 | gggggCTggTTTCAgAATA | CTCCTTgTCCTCgTCTgCAC |
| CCL20 | ATgTgCTgTACCAAgAgTTT | CAAgTCTgTTTTggATTTgC |
| CCL22 | ggAggCAAAgAgTAgggTgTAAT | TCAgCCAgAAAggCATAgATA |
| CSF-1 | CCAggAACAgTTgAAAgATCCA | TTATCTCTgAAgCgCATggTgT |
| CSF-2 | ggCCAgCCACTACAAgCAgCACT | CAAAggggATgACAAgCAgAAg |
| CXCL9 | TgCAAggAACCCCAgTAgTgA | ggTggATAgTCCCTTggTTgg |
| CXCL10 | gTggCATTCAAggAgTACCTC | TgATggCCTTCgATTCTggATT |
| CXCL11 | CCTggggTAAAAgCAgTgAA | TgggATTTAggCATCgTTgT |
| CXCL12 | TgCCAgAgCCAACgTCA | CAgCCgggCTACAATCTgAA |
| MMP-1 | AAgCCAgAgCTgTgCAgATgAgTA | TgTCCTgCAgCCACTggTTC |
| MMP-2 | CCCCAAAACggACAAAgAg | CTTCAgCACAAACAggTTgC |
| MMP-3 | CgCCTgTCTgAAgATgATATAAAT | CTgACAgCATCAAAggACAA |
| MMP-9 | gAACCAATCTCACCgACAgg | gCCACCCgAgTgTAACCATA |
| IL-6 | ACTCACCTCTTCAGAACGAATTG | CCATCTTTGGAAGGTTCAGGTTG |
| IL-8 | TTTTGCCAAGGAGTGCTAAAGA | AACCCTCTGCACCCAGTTTTC |
| IL-10 | CATAAATTAgAggTCTCCAAAATCg | AAggggCTgggTCAgCTAT |
| IL-1α | ggTTgAgTTTAAgCCAATCCA | TgCTgACCTAggCTTgATgA |
| IL-1β | TACCTgTCCTgCgTgTTgAA | TCTTTgggTAATTTTTgggATCT |
| OCT4 | ggCTTCAgACTTCgCCTCC | AACCTgAggTCCACAgTATgC |
| NANOG | TCTTCCTggTCCCCACAgTTT | gCAAgAATAgTTCTCgggATgAA |
| ARG2 | ACCTgATAgTgAATCCACgCT | CATgggCATCAACCCAgAC |
| YPEL3 | gTgCggATTTCAAAgCCCAAg | CCCACgTTCACCACTgAgTT |
| GAPDH | GACAGTCAGCCGCATCTTCTT | AATCCGTTGACTCCGACCTTC |
